# Markers of Infection-Mediated Cardiac Damage in Influenza and COVID-19

**DOI:** 10.3390/pathogens11101191

**Published:** 2022-10-16

**Authors:** Francesco Robert Burkert, Lukas Lanser, Alex Pizzini, Rosa Bellmann-Weiler, Günter Weiss

**Affiliations:** Department of Internal Medicine II, Medical University of Innsbruck, 6020 Innsbruck, Austria

**Keywords:** influenza, COVID-19, troponin-T, creatine kinase

## Abstract

Introduction: Influenza and the coronavirus disease 2019 (COVID-19) are two potentially severe viral infections causing significant morbidity and mortality. The causative viruses, influenza A/B and the severe acute respiratory syndrome coronavirus 2 (SARS-CoV2) can cause both pulmonary and extra-pulmonary disease, including cardiovascular involvement. The objective of this study was to determine the levels of cardiac biomarkers in hospitalized patients infected with influenza or COVID-19 and their correlation with secondary outcomes. Methods: We performed a retrospective comparative analysis of cardiac biomarkers in patients hospitalized at our department with influenza or COVID-19 by measuring high-sensitivity troponin-T (hs-TnT) and creatinine kinase (CK) in plasma. Secondary outcomes were intensive care unit (ICU) admission and all-cause in-hospital mortality. Results: We analyzed the data of 250 influenza patients and 366 COVID-19 patients. 58.6% of patients with influenza and 46.2% of patients with COVID-19 presented with increased hs-TnT levels. Patients of both groups with increased hs-TnT levels were significantly more likely to require ICU treatment or to die during their hospital stay. Compared with COVID-19, cardiac biomarkers were significantly higher in patients affected by influenza of all age groups, regardless of pre-existing cardiovascular disease. In patients aged under 65 years, no significant difference in ICU admission and mortality was detected between influenza and COVID-19, whereas significantly more COVID-19 patients 65 years or older died or required intensive care treatment. Conclusions: Our study shows that increased cardiac biomarkers are associated with higher mortality and ICU admission in both, influenza and SARS-CoV-2-infected patients. Cardiac biomarkers are higher in the influenza cohort; however, this does not translate into worse outcomes when compared with the COVID-19 cohort.

## 1. Introduction

The common flu is caused by influenza viruses, pathogens discovered at the beginning of the 20th century [1]. This virus has continued afflicting humanity with recurrent epidemics and sometimes pandemics [2]. These viral infections seasonally reoccur, causing extensive morbidity and mortality [3]. Most viral variants initially evolve and develop in birds [4] or swine [5], and reach human hosts in a so-called species spillover event.

Individuals infected by influenza typically develop symptoms after an incubation time of 1–4 days and may report fever with chills and night sweats, malaise, myalgia, coughing, headache and loss of appetite [6]. The symptoms persist for 5–7 days and mostly resolve without the need for medical intervention [6].

However, elderly individuals or persons with serious pre-existing diseases, especially chronic lung or heart disease, diabetes or chronic kidney disease, are at risk for life-threatening influenza infections and may require hospitalization and, in some cases, intensive care treatment [7,8]. The influenza virus replicates in the epithelial cells of the lungs; however, the virus may lead to a systemic disease, potentially damaging other organs [9]. Cardiovascular involvement and potentially myocarditis or myocardial infarction are known complications in influenza infections, which can further complicate the course of the disease [10,11]. In addition to supportive measures, specific therapeutic options against influenza are limited to antivirals such as neuraminidase or endonuclease inhibitors, which reduce the duration of disease by 1–2 days [12]. As prevention, vaccination with yearly adaptation to circulating viral strains is available with varying efficacy against infection or severe disease [13]. In addition, several different types of influenza A and B viruses occur during a typical epidemic season [6], and infections with one influenza type do not provide cross-reactive immune protection, thus repetitive influenza infections can occur within one season [14]. Furthermore, protective immunity against a specific influenza strain is short lasting, and due to constant antigenic modifications of influenza viruses repeated infections in consecutive epidemic seasons may occur [15,16].

The severe acute respiratory syndrome coronavirus 2 (SARS-CoV-2) was identified at the end of 2019 as the causal agent for the coronavirus disease 2019 (COVID-19) [17]. This viral infection easily spreads from person to person through respiratory droplets and has caused a pandemic starting in 2020 [18]. Following infection, the virus gains access to vulnerable cells through the angiotensin-converting enzyme 2 (ACE-2) receptor [19]. The primary targets are the lungs and the gastrointestinal system; therefore, affected patients often develop cough, dyspnea and airway congestion, as well as nausea, diarrhea, and characteristic anosmia and dysgeusia. Systemic inflammation further causes fever, myalgia, asthenia and cephalea, with some individuals reporting nothing more than a flu-like infection [18,20]. Patients with specific risk factors and/or who are unable to clear the infection may eventually develop a hyperinflammatory syndrome resulting in massive pulmonary inflammation and hypoxic respiratory insufficiency, requiring hospital admission and oxygen therapy, or even invasive mechanical ventilation [21]. Various vaccines are available to combat the spread and severity of COVID-19; however, the containment of the pandemic has remained challenging [22].

While the lung remains the most frequent target for SARS-CoV-2, the widespread availability of ACE-2 receptors allows the virus to invade and damage many other organs [23]. Moreover, systemic inflammation also contributes to widespread organ damage with potential progression to multi-organ dysfunction syndrome [24]. Accordingly, cardiac involvement with myocarditis or acute cardiac failure has been described [25]. Cardiac damage may be primarily caused by the infection, as viral particles may invade the myocardium and cause inflammation, or secondary, due to the pro-thrombotic effects of infection triggered immune activation and occlusion of coronary arteries resulting in myocardial infarction [26].

The aim of the present study was to evaluate pathological alterations of cardiac biomarkers among patients with influenza or COVID-19 during hospital stay and to study their correlation with intensive care unit (ICU) admission and early mortality. The novelty of our study lies in the direct comparison between these two infections, whereas previous studies analyzed the role played by cardiac biomarkers in each individual disease [27,28,29,30].

## 2. Methods

We conducted a retrospective virtual chart review of all patients admitted to the Innsbruck University Hospital between January 2012 and March 2019 because of severe influenza A or B. The comparison cohort consisted of COVID-19-positive patients admitted to the same hospital between January 2020 and February 2021. Inclusion criteria were age above 18, a positive polymerase chain reaction (PCR) for influenza or COVID-19, and a necessity for in-hospital management. Exclusion criteria were under eighteen years of age and outpatient treatment.

Patient data extracted included age, gender and a history of cardiovascular disease defined by the presence of coronary heart disease and or chronic heart failure as described by the European society of cardiology guidelines [31]. Registered outcomes were recorded as intensive care treatment, as well as all-cause in-hospital mortality. We extracted data on kidney function including serum creatinine and estimated glomerular filtration rate (eGFR), and cardiac biomarkers including high sensitivity troponin-T (hs-TnT) and creatine kinase (CK) in sequential analyses, using the highest value for statistical computations. If multiple hs-TnT values were detected, the difference between the highest and lowest value was calculated (Δhs-TnT) and used for further statistical analysis. The used hs-TnT assay was “Elecsys^®^ high-sensitive Troponin T” by Roche. The upper limit of normal for hs-TnT was 14 ng/L.

The study was retrospective in nature; therefore, no informed consent was necessary; the data collection was executed after approval by the local ethic committee (study numbers EK-1167/2020 and AN2017-0054 371/4.10).

We analyzed variables for normal distribution, if present we tested for correlation using one-way ANOVA. If no normal distribution was present, we applied the Kruskal–Wallis test by ranks.

All statistical analyses were run on R Statistics v. 4.1.2. Pictures were generated using SPSS v. 27, and tables were produced with R and Microsoft Excel 2019.

## 3. Results

We retrospectively analyzed 616 hospitalized patients, of whom 250 had been infected with influenza and 366 with COVID-19 (Table 1). Patients with influenza were on average 65.9 (SD 18.5) years old, and 114 (45.6%) patients were women. COVID-19 patients had a mean age of 63.4 (SD 17.6) years, and 139 (38.1%) were women. 134 patients (21.8%) had a history of chronic heart disease.

While mean ages for both patient groups were not significantly different, significantly more influenza patients were older than 65 years (influenza: 160 or 64.0% vs. COVID-19: 187 or 51.1%; *p* = 0.002). 

The mean eGFR calculated with the modification of diet in renal disease (MDRD)-the formula was slightly lower for influenza patients (influenza: 68.1 mL/min, SD 27.7 vs. COVID-19: 73.2 mL/min, SD 31.3; *p* = 0.046) and a higher percentage of influenza patients had a history of chronic heart disease (Table 1).

Median hs-TnT values were significantly higher in influenza patients (influenza: 17.6 ng/L, IQR 8.0–38.0 vs. COVID-19: 11.8 ng/L, IQR 5.9–27.8; *p* = 0.003), which also held true for the calculation of Δhs-TnT (influenza: 8.0 ng/L, IQR 2.8–31.2 vs. COVID-19: 3.6 ng/L, IQR 2.0–10.8; *p* = 0.001, Table 1). 

We also detected statistically significant differences in mean CK concentrations between the two groups (influenza: 144.0 U/L, IQR 76.0–309.0 vs. COVID-19: 111.5 U/L, IQR 55.2–222.7; *p* = 0.001). Of note, influenza and COVID-19 patients who were admitted to the ICU or who died had significantly higher hs-TnT levels. 

Hs-TnT values were available for 514 of 616 (83.4%) patients. Two hundred and sixty-seven patients had hs-TnT values above the upper limit of normal (ULN), 140 of 239 (58.6%) with influenza and 127 of 275 (46.2%) with COVID-19 (Table 2). When compared with patients with normal hs-TnT, more patients with values above the ULN of 14 ng/L died (41 or 15.6% vs. 5 or 2.0%; *p* < 0.001) or required intensive care treatment (69 or 25.8% vs. 43 or 17.4%; *p* = 0.02). 

Of the 41 patients with hs-TnT above the ULN and who died, significantly more were infected with COVID-19 (influenza: 15 or 10.7% vs. COVID-19: 26 or 20.5%; *p* = 0.03, Table 3). The same was also true for those subjects who were admitted to the ICU (Influenza: 23 or 16.4% vs. COVID-19: 46 or 36.2%; *p* < 0.001).

**Figure 1:**
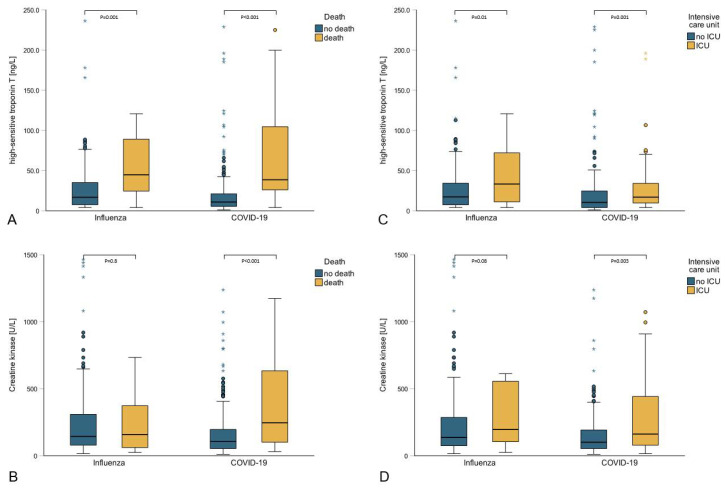
Boxplot showing comparison of ICU admission and in-hospital mortality between influenza and COVID-19 patients stratified by hs-TnT and CK. (**A**): comparison in mortality between influenza and COVID-19 stratified by hs-TnT. (**B**): comparison in mortality between influenza and COVID-19 stratified by CK. (**C**): comparison in ICU admission between influenza and COVID-19 stratified by hs-TnT. (**D**): comparison in ICU admission between influenza and COVID-19 stratified by CK. * = extreme outlier.

Fifty-eight patients died during their hospital stay (Table 4). When compared with survivors significant differences in age (survival: 63.1 years, SD 17.9 vs. death: 77.5 years, SD 12.6; *p* < 0.001), eGFR (survival: 72.5 mL/min, SD 29.5 vs. death: 55.4 mL/min, SD 28.8; *p* < 0.001), hs-TnT (survival: 12.7 ng/L, IQR 6.3–27.8 vs. death: 40.2 ng/L, IQR 24.6–89.7; *p* < 0.001, Figure 1A), Δhs-TnT (survival: 4.2 ng/L, IQR 2–13.8 vs. death: 25.7 ng/L, IQR 7.5–51.7; *p* < 0.001), CK (survival: 123 U/L, IQR 64.2–241.2 vs. death: 206.0 U/L, IQR 67.0–525.0; *p* = 0.01, Figure 1B) and ICU-admission (survival: 103 or 18.5% vs. death: 19 or 32.8%; *p* = 0.01) were detected.

The 122 patients admitted to the ICU (Table 5) had higher concentrations of hs-TnT (no ICU: 13.0 ng/L, IQR 6.0–29.4 vs. ICU: 18.3 ng/L, IQR 10.0–53.5; *p* = 0.001, Figure 1C) and CK (no ICU: 117.0 U/L, IQR 64.0–226.0 vs. ICU: 164.0 U/L, IQR 80.7–458.5; *p* = 0.004, Figure 1D), as well as higher Δhs-TnT (no ICU: 3.3 ng/L, IQR 1.7–10.8 vs. ICU: 10.0 ng/L, IQR 3.9–40.1; *p* < 0.001) and mortality (no ICU: 39 or 7.9% vs. ICU: 19 or 15.6%; *p* = 0.01).

While more influenza patients did have a history of cardiovascular disease (influenza: 92 or 36.8% vs. COVID-19: 42 or 14.3%; *p* < 0.001, Table 1), the subgroup analysis for patients with no known heart disease in their medical history still revealed a statistically significant difference in hs-TnT, with mean values being higher in patients with influenza, (influenza: 15.7 ng/L, IQR 6.6–33.0 vs. COVID-19: 10.6 ng/L, IQR 5.3–21.1; *p* = 0.02), Δhs-TnT (influenza: 6.4 ng/L, IQR 2.2–20.1 vs. COVID-19: 3.2 ng/L, IQR 1.8–8.3; *p* = 0.007) and CK (influenza: 154.0 U/L, IQR 81.0–375.0 vs. COVID-19: 109.0 U/L, IQR 53.7–197.0; *p* < 0.001). No significant differences were found in these parameters for the patients without preexisting cardiovascular disease.

In the comprehensive analysis involving all patients, we detected no significant difference in mortality between influenza and COVID-19 (influenza: 19 or 7.6% vs. COVID-19: 39 or 10.7%; *p* = 0.2, Table 1). However, admission to ICU was more frequent in COVID-19 patients (influenza: 35 or 14.0% vs. COVID-19: 87 or 23.8%; *p* = 0.003).

A further subgroup analysis for patients under 65 years of age confirmed differences in hs-TnT (influenza: 8.0 ng/L, IQR 4.0–20.0 vs. COVID-19: 6.1 ng/L, IQR 4.0–11.4; *p* = 0.04) and Δhs-TnT (influenza: 19.8 ng/L, IQR 3.8–36.8 vs. COVID-19: 3.2 ng/L, IQR 1.8–7.9; *p* < 0.001), while patients over 65 differed prevalently in CK levels (influenza: 140.0 U/L, IQR 86–286 vs. COVID-19: 108 U/L, IQR 54.7–245.5; *p* = 0.007). Of note, significantly more COVID-19 patients older than 65 years of age died (influenza: 16 or 10% vs. COVID-19: 32 or 18.2%; *p* = 0.03) or were admitted to the ICU (influenza: 17 or 10.6% vs. COVID-19: 38 or 20.3%; *p* = 0.01). However, we detected no difference in ICU admission and mortality for patients under 65 between influenza and COVID-19.

## 4. Discussion

Cardiac involvement has been demonstrated for influenza [32,33] but also for COVID-19 [34].

In this retrospective comparative study, we attempted to perform a comparative analysis of the difference in laboratory markers for cardiac involvement in these two infections. We found that, based on our data, influenza infections more frequently resulted in a pathologic increase in cardiac biomarkers as compared with COVID-19. Moreover, mean hs-TnT levels in affected patients were also higher in influenza subjects as compared with COVID-19 patients. We detected this effect throughout all our subgroup analyses, independent of pre-existing cardiovascular disease. Increased hs-TnT was associated with a significantly increased risk for ICU admission and higher in-hospital mortality for both influenza and COVID-19, but the higher levels in the influenza cohort did not result in more ICU admission or mortality than in the COVID-19 cohort.

Significantly more COVID-19 patients over 65 years of age died or required ICU admission during their hospital stay when compared with influenza which was true for groups with normal or increased hs-TnT. In contrast, in patients under 65 years of age, no difference in death or ICU admission was detected among influenza and COVID-19 patients, once again showing the importance of age as a risk factor for a severe disease course of COVID-19 [35]. The healthcare system was not overloaded during the two periods investigate; therefore, all patients received intensive care treatment according to the standard of care, thereby reducing the risk of triage-related selection bias.

A possible cause of cardiac damage in COVID-19 and influenza is hypercoagulability leading to the emergence of microthrombi potentially causing disruption of cardiac microcirculation [36,37,38]. In addition, both infections may become invasive in the heart causing myocarditis and immune cell infiltration with subsequent inflammation [39,40]. Interestingly, dietary habits, specifically the consumption of a high-fat diet, may contribute to cardiac damage in influenza infection [41]. Cardiac involvement may resolve with improvement in the infection but may also persist for a prolonged period, thereby being associated with reduced cardiovascular performance post-infection [42].

Our study has some limitations. Specific analysis for influenza subtypes, which could be of interest, as influenza A and B may differently affect the myocardium, could not be performed due to incomplete data. The study only analyzed hospitalized patients, not taking into account patients that qualified for out-of-hospital treatment, which may have influenced the data. Only data regarding pre-existing cardiovascular disease was collected; therefore, further causes of hs-TnT elevation such as drug toxicity, embolic disease or acute neurological disease may have acted as undetected confounders. It is also not possible to determine whether hs-TnT increased in the context of other vascular events such as non-ST-elevation myocardial infarction, although influenza infections can be associated with myocardial ischemia and arrhythmia [33]. The higher hs-TnT levels found in the influenza group may also be partially explained by the larger representation of patients over 65 years and slightly lower eGFR compared with COVID-19.

It is important to note that the study periods for influenza and COVID-19 data were asynchronous and this may be a confounder. Of note, we recorded very few influenza cases at our institution after the onset of COVID-19 probably due to social distancing and mask usage but also unweighted diagnostics in primary care and by governmental diagnostic services which focused on the identification of SARS-CoV2 infection by PCR testing thereby ignoring other causes of respiratory infections.

Our study period predates the emergency use authorization of antivirals against SARS-CoV-2 and data regarding specific antiviral therapy was not collected for the influenza cohort. We acknowledge that this may act as a further confounder, as the patients afflicted with influenza may have experienced better outcomes in part due to the availability of targeted antiviral therapy with neuraminidase inhibitors.

Furthermore, the data collected for COVID-19 was limited to two winter seasons without data regarding the most recent Omicron variant, and the data predate the introduction of vaccines against COVID-19. No data was collected regarding the vaccine status of patients affected by influenza. Finally, due to the retrospective nature of the study, Δhs-TnT data could only be collected for the included patients who happened to receive multiple blood tests during their hospital stay.

This research helps in shedding light on important differences in cardiac involvement for two major contemporary viral diseases. Future investigations with direct comparison of influenza and COVID-19 cases in hospitalized patients within one season are needed to further elucidate the significance of these findings in clinical practice. However, this will depend on the respective circulating SARS-CoV-2 and influenza subtypes, the clinical pathologies they cause changing and immune protection originating from vaccine coverage, efficacy and previous infections.

## 5. Conclusions

Our retrospective comparative analysis of cardiac biomarkers in a cohort of hospital-admitted patients demonstrated that a high percentage of patients with influenza or COVID-19 had increased levels of the cardiac biomarkers hs-TnT, and CK. Relatively more patients with influenza than COVID-19 had pathologic levels of these biomarkers and the mean values were also higher in influenza as compared with COVID-19 irrespective of a history of cardiovascular disease.

While elevated cardiac biomarkers were associated with higher ICU admission and in-hospital mortality rate, no differences were seen in that respect between influenza and COVID-19 cohorts. However, in general patients above 65 years had a higher risk of death from COVID-19 as compared with subjects infected with influenza.

Further investigations are warranted to clarify the pathogenic mechanisms underlying these findings, as well as their significance in the clinical management of these patients.

## Figures and Tables

**Table 1:** Comparison between influenza and COVID-19 in the patient cohort.

	Overall	Influenza	COVID-19	*p*
n	616	250	366	
Patient age, years (mean (SD))	64.43 (18.00)	65.92 (18.49)	63.41 (17.61)	0.089
Age over 65 (%)	347 (56.3)	160 (64.0)	187 (51.1)	0.002
Patient sex = woman (%)	253 (41.1)	114 (45.6)	139 (38.1)	0.063
Creatinine, mg/dL (median [IQR])	0.98 [0.80, 1.21]	1.01 [0.83, 1.22]	0.94 [0.79, 1.19]	0.156
EGFR, mL/min (mean (SD))	70.91 (29.81)	68.08 (27.72)	73.20 (31.27)	0.046
Hs-TnT, ng/L (median [IQR])	15.20 [6.60, 32.88]	17.60 [8.00, 38.05]	11.80 [5.90, 27.80]	0.003
Hs-TnT over 14 ng/L (%)	267 (51.9)	140 (58.6)	127 (46.2)	0.005
Δhs-TnT, ng/L (median [IQR])	4.90 [2.08, 18.05]	8.00 [2.85, 31.25]	3.60 [2.00, 10.80]	0.001
Creatine kinase, U/L (median [IQR])	126.00 [64.50, 269.00]	144.00 [76.00, 309.00]	111.50 [55.25, 222.75]	0.001
In-hospital mortality (%)	58 (9.4)	19 (7.6)	39 (10.7)	0.202
ICU admittance (%)	122 (19.8)	35 (14.0)	87 (23.8)	0.003
Chronic heart disease (%)	134 (24.6)	92 (36.8)	42 (14.3)	<0.001

Test for variables with normal distribution: one-way ANOVA. Test for variables with non-normal distribution: Kruskal–Wallis test by ranks.

**Table 2:** Comparison between patients above and below the 14 ng/L hs-TnT cutoff.

	Overall	Hs-TnT under 14 ng/L	Hs-TnT over 14 ng/L	*p*
*n*	514	247	267	
Patient age, years (mean (SD))	64.49 (18.17)	54.54 (17.31)	73.72 (13.49)	<0.001
Patient sex = woman (%)	218 (42.4)	117 (47.4)	101 (37.8)	0.029
Creatinine, mg/dL (median [IQR])	0.96 [0.79, 1.22]	0.87 [0.73, 1.00]	1.12 [0.89, 1.54]	<0.001
EGFR, mL/min (mean (SD))	71.21 (30.13)	85.64 (27.41)	57.92 (26.18)	<0.001
Hs-TnT, ng/L (median [IQR])	15.20 [6.60, 32.88]	6.50 [4.00, 9.55]	31.90 [20.15, 55.60]	<0.001
Creatine kinase, U/L (median [IQR])	127.00 [67.00, 272.00]	109.00 [63.00, 181.00]	154.00 [76.00, 406.00]	<0.001
In-hospital mortality (%)	46 (8.9)	5 (2.0)	41 (15.6)	<0.001
ICU admittance (%)	112 (21.8)	43 (17.4)	69 (25.8)	0.021
Chronic heart disease (%)	122 (26.6)	34 (15.5)	88 (36.8)	<0.001

Data available for 514 patients. Test for variables with normal distribution: one-way ANOVA. Test for variables with non-normal distribution: Kruskal–Wallis test by ranks.

**Table 3:** Comparison between influenza and COVID-19 patients with hs-TnT above 14 ng/L.

	Overall	Influenza	COVID-19	*p*
*n*	267	140	127	
Patient age, years (mean (SD))	73.72 (13.49)	72.87 (14.73)	74.67 (11.95)	0.279
Patient sex = woman (%)	101 (37.8)	64 (45.7)	37 (29.1)	0.005
Creatinine, mg/dL (median [IQR])	1.12 [0.89, 1.54]	1.07 [0.90, 1.44]	1.16 [0.88, 1.65]	0.341
EGFR, mL/min (mean (SD))	57.92 (26.18)	58.23 (25.08)	57.58 (27.44)	0.841
Hs-TnT, ng/L (median [IQR])	31.90 [20.15, 55.60]	33.55 [21.95, 58.35]	29.40 [19.20, 53.30]	0.343
Creatine kinase, U/L (median [IQR])	154.00 [76.00, 406.00]	157.00 [86.00, 455.00]	150.50 [63.50, 384.00]	0.239
In-hospital mortality (%)	41 (15.4)	15 (10.7)	26 (20.5)	0.027
ICU admittance (%)	69 (25.8)	23 (16.4)	46 (36.2)	<0.001
Chronic heart disease (%)	88 (36.8)	64 (45.7)	24 (24.2)	0.001

Test for variables with normal distribution: one-way ANOVA. Test for variables with non-normal distribution: Kruskal–Wallis test by ranks.

**Table 4:** Comparison of patients who died and who survived during their hospital stay.

	Overall	Survival	Death	*p*
*n*	616	558	58	
Patient age, years (mean (SD))	64.43 (18.00)	63.07 (17.94)	77.52 (12.58)	<0.001
Creatinine, mg/dL (median [IQR])	0.98 [0.80, 1.21]	0.95 [0.79, 1.16]	1.30 [0.96, 1.65]	<0.001
EGFR, mL/min (mean (SD))	70.91 (29.81)	72.51 (29.48)	55.38 (28.82)	<0.001
Hs-TnT, ng/L (median [IQR])	15.20 [6.60, 32.88]	12.75 [6.30, 27.80]	40.25 [24.60, 89.73]	<0.001
Δhs-TnT, ng/L (median [IQR])	4.90 [2.08, 18.05]	4.25 [2.00, 13.78]	25.70 [7.50, 51.75]	<0.001
Creatine kinase, U/L (median [IQR])	126.00 [64.50, 269.00]	123.00 [64.25, 241.25]	206.00 [67.00, 525.00]	0.012
ICU admittance (%)	122 (19.8)	103 (18.5)	19 (32.8)	0.009
Chronic heart disease (%)	134 (24.6)	118 (23.8)	16 (33.3)	0.143

Test for variables with normal distribution: one-way ANOVA. Test for variables with non-normal distribution: Kruskal–Wallis test by ranks.

**Table 5:** Comparison between patients who required intensive care treatment and those who did not.

	Overall	No ICU Admission	ICU Admission	*p*
*n*	616	494	122	
Patient age, years (mean (SD))	64.43 (18.00)	65.21 (18.85)	61.27 (13.70)	0.03
Creatinine, mg/dL (median [IQR])	0.98 [0.80, 1.21]	0.97 [0.80, 1.16]	1.00 [0.79, 1.43]	0.206
EGFR, mL/min (mean (SD))	70.91 (29.81)	71.14 (29.21)	70.06 (32.08)	0.729
Hs-TnT, ng/L (median [IQR])	15.20 [6.60, 32.88]	13.00 [6.00, 29.40]	18.35 [10.05, 53.50]	0.001
Δhs-TnT, ng/L (median [IQR])	4.90 [2.08, 18.05]	3.30 [1.75, 10.80]	10.00 [3.90, 40.10]	<0.001
Creatine kinase, U/L (median [IQR])	126.00 [64.50, 269.00]	117.00 [64.00, 226.00]	164.50 [80.75, 458.50]	0.004
In-hospital mortality (%)	58 (9.4)	39 (7.9)	19 (15.6)	0.009
Chronic heart disease (%)	134 (24.6)	118 (26.5)	16 (16.2)	0.031

Test for variables with normal distribution: one-way ANOVA. Test for variables with non-normal distribution: Kruskal–Wallis test by ranks.

## Data Availability

Not applicable.
